# Supplementary material for: Impact of keratocyte differentiation on corneal opacity resolution and visual function recovery in male rats
Source: Nat Commun. 2024 Jun 11;15:4959. doi: 10.1038/s41467-024-49008-3 (PMC11166667; doi:10.1038/s41467-024-49008-3)
Supplement: Supplementary file 1 — Supplementary Information [file 41467_2024_49008_MOESM1_ESM.pdf]

Impact of keratocyte differentiation on corneal opacity resolution and visual function recovery in male rats

Andri K. Riau[a,b,*], Zhuojian Look[a,b,*], Gary H. F. Yam[a,c], Craig Boote[d], Qian Ma[d], Evelina J. Y. Han[a], Nur Zahirah binte M. Yusoff[a], Hon Shing Ong[a,b,e], Tze-Wei Goh[a], Nuur Shahinda Humaira binte Halim[a], Jodhbir S. Mehta[a,b,e,ⱡ]

[a]Tissue Engineering and Cell Therapy Group, Singapore Eye Research Institute, Singapore 169856, Singapore
[b]Ophthalmology and Visual Sciences Academic Clinical Programme, Duke-NUS Medical School, Singapore 169857, Singapore
[c]Department of Ophthalmology, University of Pittsburgh, Pittsburgh, PA 15213, USA
[d]School of Optometry and Vision Sciences, Cardiff University, Cardiff CF24 4HQ, UK
[e]Corneal and External Eye Diseases Department, Singapore National Eye Centre, Singapore 168751, Singapore

[*]These authors contributed equally to the manuscript

[ⱡ]Corresponding author:
Prof. Jodhbir Singh Mehta
Phone: (65) 6322 8387
Email: jodhbir.s.mehta@singhealth.com.sg

SUPPLEMENTARY DISCUSSION

Cell delivery quality dictates the therapeutic efficiency of cultivated keratocytes

We observed that corneas that received poorly delivered quiescent corneal stromal keratocytes (qCSKs) had haze that persisted at almost the same level over 21 days after cell injection (Supplementary Fig. 2b, c and Supplementary Table S2). In contrast, the haze grades steadily reduced over time in the corneas that received good quality qCSK delivery, and the disparity compared to the "poor" delivery was significant at post-injection (PID) 21 (p=0.015).

Poor delivery also resulted in increasing neovascularization (NV) severity scores over time (Supplementary Fig. 2b, d and Supplementary Table 2). Some corneas in the good delivery group also developed NV in the earlier follow-up periods, which was expected due to the needle injection into the stroma, but the NV severity was reduced after 21 days. The NV score of the good delivery group was substantially lower than that of the "poor" delivery group (p=0.013). The total score, cumulative of the haze and NV scores showed that the sequelae of the irregular phototherapeutic keratectomy (IrrPTK) injury exacerbated with time when the qCSKs were poorly delivered (Supplementary Fig. 2e and Supplementary Table 2). A complete opposite trend, reflecting steady recovery, was observed when the delivery quality was good. The difference in the total score was statistically significant at PID21 (p=0.008).

Analysis of haze density and area based on in vivo confocal microscopy (IVCM) and slit-lamp biomicroscopy, respectively, allowed for a more objective assessment of the outcomes of any intrastromal cell delivery. The haze density at the IrrPTK-injury site, corresponding to the intensity of the stromal hyperreflectivity captured by IVCM in the anterior third of the corneal stroma, was higher in the corneas that received "poor" cell delivery at all follow-up time points and notably more elevated after 21 days (p=0.003) (Supplementary Fig. 2f and Supplementary Table 3). The corneal haze area was only reduced by 7.40±4.66% after 21 days in the "poor" delivery group from PID0 (Supplementary Fig. 2g and Supplementary Table 4). In contrast, the haze area steadily decreased in the good delivery group at every time point, reaching a maximum

reduction of 16.19±3.77% after 21 days. The haze area was remarkably lower than the "poor" delivery group in the last follow-up period (p=0.038).

Because the rats' central corneal thickness (CCT) varied, we normalized the thickness difference by presenting % changes in the CCT (% ΔCCT) over time from the preoperative CCT. Our results showed that the % ΔCCT of both good and poor delivery groups decreased after qCSK injection and the rate was similar up to post-injection day 7 (Supplementary Fig. 2h and Supplementary Table 5). The % ΔCCT reduction appeared to plateau after 14 days in the poor delivery group but continued a downward trend in the good delivery group.

The differentiation state determines the efficacy of cultivated keratocytes in corneal stromal cell therapy

The activated CSK (aCSK) injection resulted in mild haze formation and NV, the extent of which was only marginally higher than the qCSK injection (Supplementary Fig. 3b). We did not find any significant difference in their haze (Supplementary Fig. 3c and Supplementary Table 6), NV (Supplementary Fig. 3d and Supplementary Table 6), and total (Supplementary Fig. 3e and Supplementary Table 6) scores at any follow-up periods. SF injection resulted in a more severe haze and NV, although the NV appeared to resolve in all six rats after 21 days. Nonetheless, the total clinical score of the stromal fibroblasts (SFs) group was significantly higher than the CSKs group at the last follow-up (p=0.046).

The IVCM-based haze density analysis concurred with the slit-lamp observation, where the aCSK and qCSK injections did not result in obvious injury or corneal opacity, while the SF injection produced a mild haze that gradually increased in intensity, reaching a statistically significant level at PID21 in comparison to the CSKs group (p=0.045) (Supplementary Fig. 3f and Supplementary Table 7). The trend in the haze area followed the haze density in all three groups. The haze area became larger over time after SF injection and was significantly different from the CSKs group (p=0.002) (Supplementary Fig. 3g and Supplementary Table 8). Although we did not find significant differences in the % ΔCCT, the SF injection resulted in higher CCT changes and stayed at similar levels over 21 days (Supplementary Fig. 3h and Supplementary

Table 9). Activated CSK injection caused an increase in CCT at PID7 but the CCT was reduced in the subsequent follow-up periods, reaching 6.45±7.35% off preoperative CCT on day 21 (p=0.754 vs. CSKs group). We also found an elevation of CCT on day 7 after the qCSK injection but the resolution of the corneal edema was more rapid than the aCSK-treated corneas, achieving a CCT of -0.01±6.48% off the preoperative corneas at PID21.

SUPPLEMENTARY FIGURES

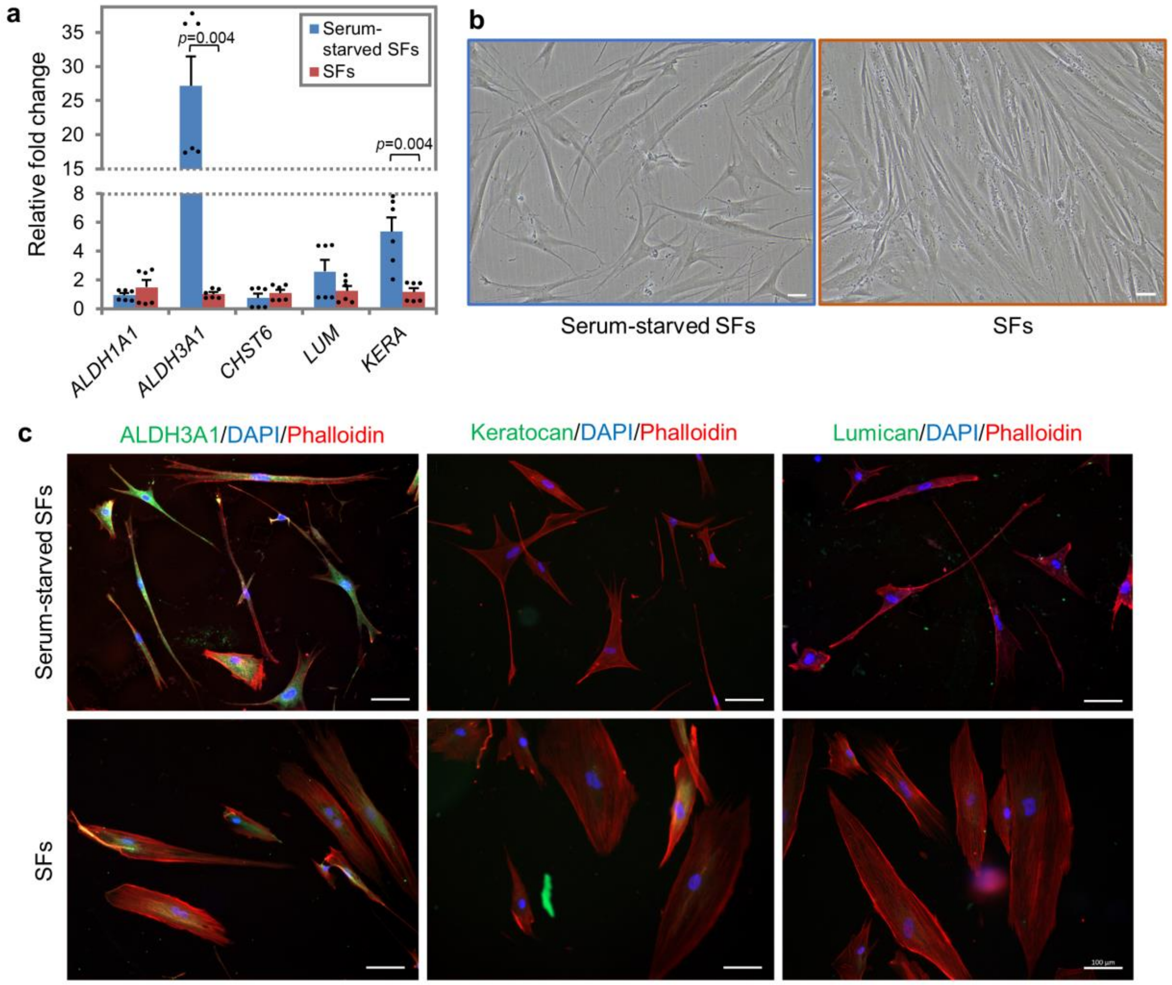

Supplementary Fig. 1. Cellular phenotypes and morphology following serum starving of corneal stromal fibroblasts.

(a) Total RNAs were isolated from stromal fibroblasts (SFs), which were expanded in the presence of 10% serum (red bars), and SFs, which were serum starved for 14 days (blue bars). The transcriptional levels of the genes were normalized internally to the level of housekeeping gene *GAPDH* and calibrated to the SFs condition. The *ALDH1A1*, *CHST6*, and *LUM* expression of serum-starved SFs mostly mirrored the SFs, except for the significant upregulation of *ALDH3A1* and *KERA*. *B3GNT7* was undetectable in both conditions. Data are presented as mean±SEM. Statistical significance was analyzed with a two-tailed Mann-Whitney U test. n=6 in each group. (b) Morphologically, serum-starved SFs demonstrated an intermediate form between control SFs and quiescent corneal stromal keratocytes (qCSKs) (see Fig. 1a for comparison). Most of these cells still possessed elongated processes akin to the

qCSKs but had pronounced cell body enlargement, bipolar orientation, and parallel arrangement—characteristics of SFs. (c) Immunofluorescence staining revealed an absence of lumican and keratocan protein expression in both serum-starved SFs and SFs, correlating with their downregulated gene expression. In contrast, ALDH3A1 protein expression was notably enhanced in the serum-deprived cells as compared to SFs, consistent with the upregulated transcription. Brightfield cellular imaging and staining were performed on 3 independent samples. Scale bars = 100 μm. Source data are provided as a Source Data file.

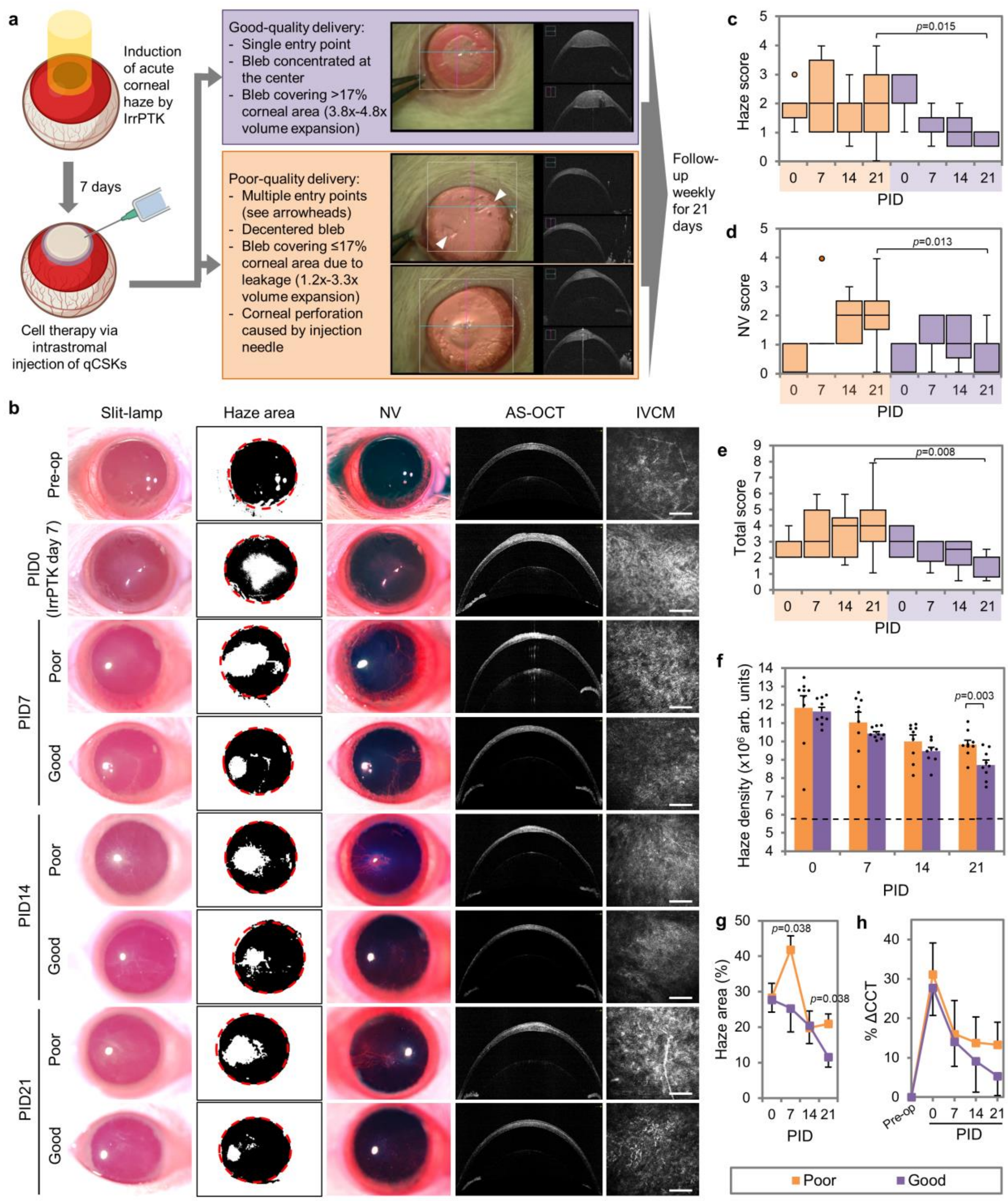

Supplementary Fig. 2. Postoperative outcomes of good and poor keratocyte delivery qualities in corneas with acute opacity.

(a) Experimental timeline and criteria of cell delivery quality. Examples of cornea appearance following good and poor quiescent corneal stromal keratocyte (qCSK) deliveries could be seen in the slit-lamp and anterior segment-optical coherence tomography (AS-OCT) images. Large bleb was normally seen following “good” delivery. Smaller bleb due to leakage and multiple injection entry points (arrowheads)

were some of the features of poor delivery quality. (b) The outcomes of injections were observed with slit-lamp, AS-OCT, and in vivo confocal microscopy (IVCM). The black and white slit-lamp images were used to aid the visualization of the haze. Red dash circles indicate the pupils. Separately, the red color of the images was enhanced to aid the visualization of blood vessels in the corneas. Further analysis of the imaging was performed by grading the haze (c) and neovascularization or NV (d) severity. (e) The total score, cumulative of the haze and NV scores, was also presented. In the box plots, the center line shows the median; box limits show the 1st and 3rd quartile; whiskers show minimum and maximum values; and points indicate outliers. Statistical significance between poor and good delivery groups was analyzed with 2-tailed Mann-Whitney U test. (f) An objective analysis was done by measuring the haze density from the IVCM images. The result agreed to the slit-lamp photography and the clinical grading. The dash line indicates the mean haze density preoperatively. (g) The haze area was measured based on the black-and-white slit-lamp images. (h) The % changes in the central corneal thickness (% ΔCCT) over time from the preoperative CCT, measured with AS-OCT. Data are presented as mean±SEM. Statistical significance between the two groups was analyzed with 2-tailed Mann-Whitney U test. PID = post-injection day. PID0 = post-irregular phototherapeutic keratectomy (IrrPTK) day 7. The poor and good delivery groups are represented by orange and purple, respectively in the box plots, bar graphs, and line graphs. n=9 in each group. Scale bars = 100 µm. Source data are provided as a Source Data file.

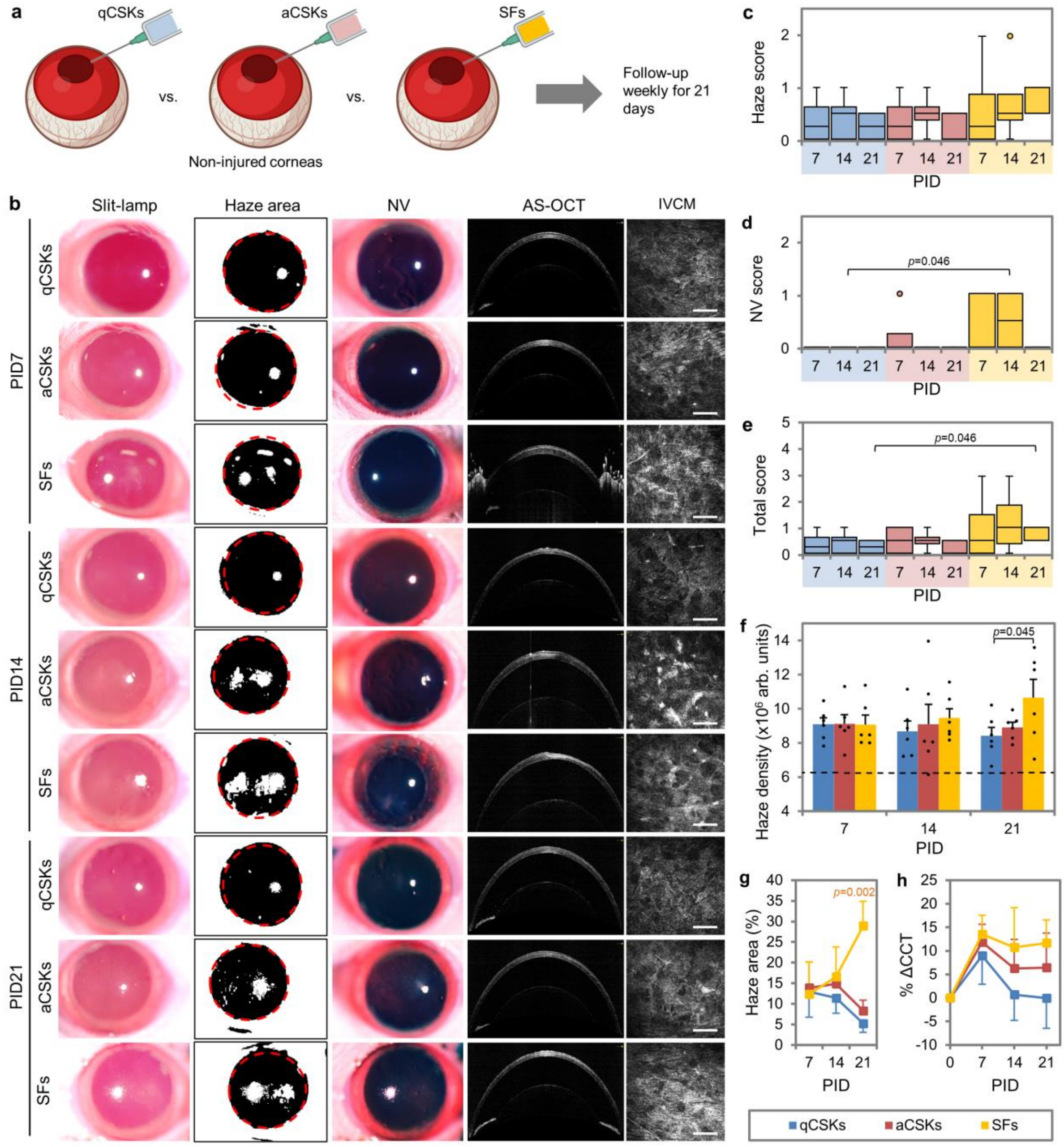

Supplementary Fig. 3. Postoperative outcomes of quiescent corneal stromal keratocyte, activated keratocyte, and stromal fibroblast injections in naïve corneas.

(a) Experimental setup and timeline. (b) The outcomes of the cell injections were observed with slit-lamp photography, anterior segment-optical coherence tomography (AS-OCT), and in vivo confocal microscopy (IVCM). The black and white slit-lamp images were used to aid the visualization of the haze. Red dash circles indicate the pupils. Separately, the red color of the images was enhanced to aid the visualization of blood vessels in the corneas. The corneal imaging showed a similarly mild haze

formation and neovascularization (NV) over 21 days between quiescent corneal stromal keratocytes (qCSKs) and activated CSKs (aCSKs) groups. Stromal fibroblasts (SFs) injection resulted in a slightly more severe haze and NV. Further analysis of the imaging outcomes was performed by grading the haze (c) and NV (d) severity. (e) The total score was presented to show the overall state of the cornea. In the box plots, the center line shows the median; box limits show the 1st and 3rd quartile; whiskers show minimum and maximum values; and points indicate outliers. Statistical significance between groups was analyzed with Kruskal-Wallis, followed by the post-hoc Dunn-Bonferroni test. (f) The haze density was measured from the IVCM images of the anterior third of the stroma. The result was consistent with the slit-lamp photography and the clinical grading. The dash line indicates the preoperative light reflectivity level. (g) The haze area was measured based on the black-and-white slit-lamp images. (h) The % changes in the central corneal thickness (% ΔCCT) over time from the preoperative CCT revealed a continued reduction of corneal thickness after post-injection day (PID) 7 in the qCSKs and aCSKs groups, but not the SFs group. Data are presented as mean±SEM. Statistical significance between groups was analyzed with one-way ANOVA, followed by a post-hoc Tukey test. The qCSKs, aCSKs, and SFs groups are represented by blue, red, and yellow, respectively in the box plots, bar graphs, and line graphs. n=6 in each group. Scale bars = 100 µm. Source data are provided as a Source Data file.

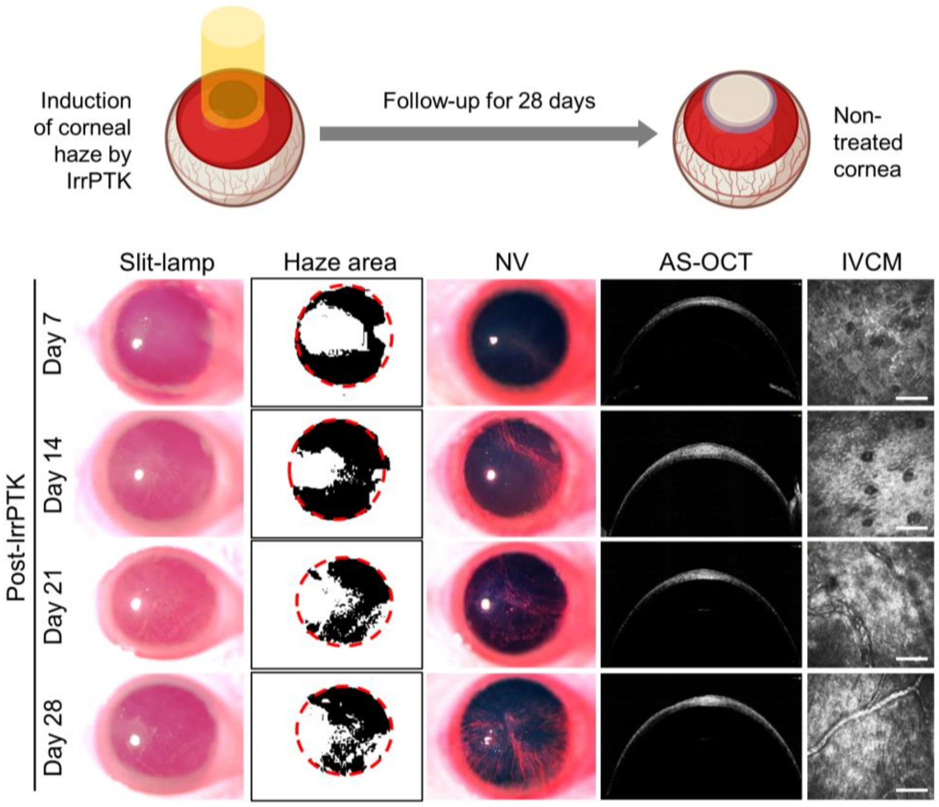

Supplementary Fig. 4. Follow-up examination outcomes of non-treated irregular phototherapeutic keratectomy-injured rats over 28 days.

The corneal examination included slit-lamp photography, in vivo confocal microscopy (IVCM), and anterior segment-optical coherence tomography (AS-OCT). The haze developed and persisted over 28 days post-laser injury in the rat when no treatment was administered. Neovascularization (NV) developed later and became more severe over time. In the treatment arm, corneal cell therapy was given 7 days after irregular phototherapeutic keratectomy (IrrPTK) in the acute opacity model or 28 days in the chronic opacity model. Red dash circles indicate the pupils. Scale bars = 100µm.

SUPPLEMENTARY TABLES

Supplementary Table 1. Fold changes of corneal stromal keratocyte-associated gene expression in cultured stromal cells relative to the native corneal stroma (n=3 in each group).

Gene	qCSKs	aCSKs	p vs. qCSKs	SFs	p vs. qCSKs
ALDH1A1	0.322±0.056	0.099±0.049	*0.047*	0.034±0.016	*0.016*
ALDH3A1	0.760±0.148	0.086±0.032	*0.010*	0.006±0.002	*0.006*
LUM	0.564±0.185	0.036±0.025	0.067	0.004±0.003	*0.036*
KERA	0.786±0.161	0.142±0.016	*0.017*	0.029±0.015	*0.008*
CHST6	0.503±0.204	0.039±0.010	0.137	0.011±0.002	0.114
B3GNT7	0.183±0.037	0.063±0.002	*0.045*	0.003±0.001	*0.007*
COL8A2	0.337±0.096	0.082±0.031	0.092	0.025±0.007	*0.040*

Source data are provided as a Source Data file

Supplementary Table 2. Clinical haze and neovascularization scores following good versus poor quality keratocyte injections in laser-injured rat corneas (n=9 in each group).

	PID0*	p	PID7	p	PID14	p	PID21	p
Haze score								
Good	2 (2, 3)	0.154	1 (1, 1)	0.051	1 (0.5, 1)	0.156	1 (0.5, 1)	*0.015*
Poor	2 (2, 2)		2 (1, 3)		2 (1, 2)		2 (1, 3)	
NV score								
Good	1 (0, 1)	0.999	1 (1, 2)	0.695	1 (1, 2)	0.067	1 (0, 1)	*0.013*
Poor	1 (0, 1)		1 (1, 1)		2 (1, 2)		2 (2, 2)	
Total score (haze + NV)								
Good	3 (2, 4)	0.489	3 (2, 3)	0.136	2.5 (2, 3)	0.077	2 (1, 2)	*0.008*
Poor	2 (2, 3)		3 (2, 5)		4 (2, 4)		4 (3, 4)	

Scores are expressed in median (Q1, Q3)

PID = post-injection day

*PID0 occurred 7 days after IrrPTK

NV = neovascularization

Source data are provided as a Source Data file

Supplementary Table 3. Corneal haze density (IntegratedDensity) following good versus poor quality keratocyte injections in laser-injured rat corneas (n=9 in each group).

	PID0* ($x10^6$ arb.units)	p	PID7 ($x10^6$ arb.units)	p	PID14 ($x10^6$ arb.units)	p	PID21 ($x10^6$ arb.units)	p
Good	11.62± 0.66	0.122	10.43± 0.55	0.085	9.46± 0.34	0.145	8.70± 0.24	*0.003*
Poor	11.83± 0.24		11.03± 0.10		9.99± 0.22		9.83± 0.27	

PID = post-injection day

*PID0 occurred 7 days after IrrPTK

Source data are provided as a Source Data file

Supplementary Table 4. Percent corneal haze area following good versus poor quality keratocyte injections in laser-injured rat corneas (n=9 in each group).

	PID0* (%)	p	PID7 (%)	p	PID14 (%)	p	PID21 (%)	p
Good	27.75± 3.98	0.757	25.33± 3.87	*0.038*	20.39± 4.78	0.895	11.56± 2.70	*0.038*
Poor	28.37± 3.52		41.83± 6.68		19.79± 5.00		20.96± 2.87	

PID = post-injection day

*PID0 occurred 7 days after IrrPTK

Source data are provided as a Source Data file

Supplementary Table 5. Percent change of central corneal thickness from preoperation following good versus poor quality keratocyte injections in laser-injured rat corneas (n=9 in each group).

	PID0* (%)	p	PID7 (%)	p	PID14 (%)	p	PID21 (%)	p
Good	27.79± 7.08	0.566	14.10± 6.30	0.895	9.16± 7.87	0.965	5.34± 4.94	0.354
Poor	31.13± 8.03		16.02± 8.47		13.82± 6.48		13.28± 5.76	

PID = post-injection day

*PID0 occurred 7 days after IrrPTK

Source data are provided as a Source Data file

Supplementary Table 6. Clinical haze and neovascularization scores following cell injections in naïve rat corneas (n=6 in each group).

	**PID7**	**p vs. qCSKs**	**PID14**	**p vs. qCSKs**	**PID21**	**p vs. qCSKs**
<u>Haze score</u>						
qCSKs	0.25 (0, 0.5)	-	0.5 (0.125, 0.5)	-	0.25 (0, 0.5)	-
aCSKs	0.25 (0, 0.5)	0.999	0.5 (0.5, 0.5)	0.999	0.5 (0.125, 0.5)	0.999
SFs	0.25 (0, 0.5)	0.999	0.5 (0.5, 0.5)	0.280	0.5 (0.5, 0.875)	0.311
<u>NV score</u>						
qCSKs	0 (0, 0)	-	0 (0, 0)	-	0 (0, 0)	-
aCSKs	0 (0, 0)	0.999	0 (0, 0)	0.999	0 (0, 0)	0.999
SFs	0 (0, 0.75)	0.449	0.5 (0, 1)	<u>*0.046*</u>	0 (0, 0)	0.999
<u>Total score (haze + NV)</u>						
qCSKs	0.25 (0, 0.25)	-	0.5 (0.125, 0.5)	-	0.25 (0, 0.5)	-
aCSKs	0.5 (0.125, 0.875)	0.999	0.5 (0.5, 0.5)	0.999	0.5 (0.125, 0.5)	0.999
SFs	0. (0.125, 0.875)	0.776	1 (0.5, 1.5)	0.232	0.5 (0.5, 0.875)	<u>*0.046*</u>

Scores are expressed in median (Q1, Q3)

PID = post-injection day

NV = neovascularization

Source data are provided as a Source Data file

Supplementary Table 7. Corneal haze density (IntegratedDensity) following cell injections in naïve rat corneas (n=6 in each group).

	PID7 ($x10^6$ arb. units)	p vs. qCSKs	PID14 ($x10^6$ arb. units)	p vs. qCSKs	PID21 ($x10^6$ arb. units)	p vs. qCSKs
qCSKs	9.09±0.38	-	8.68±0.60	-	8.41±0.50	-
aCSKs	9.12±0.53	0.989	9.11±1.16	0.942	8.91±0.30	0.848
SFs	9.07±0.55	0.850	9.46±0.55	0.999	10.67±1.06	*0.045*

PID = post-injection day

Source data are provided as a Source Data file

Supplementary Table 8. Percent corneal haze area following cell injections in naïve rat corneas (n=6 in each group).

	PID7 (%)	p vs. qCSKs	PID14 (%)	p vs. qCSKs	PID21 (%)	p vs. qCSKs
qCSKs	12.88±6.14	-	11.42±3.75	-	5.21±2.09	-
aCSKs	13.85±6.28	0.994	14.83±2.43	0.876	8.30±2.57	0.846
SFs	12.35±7.83	0.998	16.57±7.20	0.742	28.96±5.98	*0.002*

PID = post-injection day

Source data are provided as a Source Data file

Supplementary Table 9. Percent change of central corneal thickness from preoperation following cell injections in naïve rat corneas (n=6 in each group).

	PID7 (%)	p vs. qCSKs	PID14 (%)	p vs. qCSKs	PID21 (%)	p vs. qCSKs
qCSKs	9.01±6.06	-	0.69±5.45	-	-0.01±6.48	-
aCSKs	11.87±3.79	0.905	6.26±6.17	0.834	6.45±7.35	0.754
SFs	13.57±4.00	0.777	10.72±8.48	0.565	11.68±4.89	0.413

PID = post-injection day

Source data are provided as a Source Data file

Supplementary Table 10. Clinical haze and neovascularization scores following injections of different cell types in rat corneas with acute haze (n=6 in each group).

	**PID0***	**p vs. qCSKs / Non-treated**	**PID7**	**p vs. qCSKs / Non-treated**	**PID14**	**p vs. qCSKs / Non-treated**	**PID21**	**p vs. qCSKs / Non-treated**
Haze score								
qCSKs	2<br>(2, 2.75)	-<br>0.999	1<br>(1, 1.75)	-<br>0.380	0.75<br>(0.5, 1)	-<br>0.327	0.5<br>(0.5, 0.5)	-<br>*0.008*
aCSKs	2<br>(2, 2)	0.999/<br>0.999	2<br>(1.25, 2)	0.999/<br>0.999	1<br>(0.625, 1.75)	0.999/<br>0.719	1<br>(0.625, 1)	0.999/<br>0.105
SFs	2<br>(2, 2.75)	0.999/<br>0.999	2.5<br>(2, 3)	0.086/<br>0.999	2<br>(1.25, 2.75)	0.172/<br>0.999	1.5<br>(1, 2)	0.105/<br>0.999
Non-treated	2<br>(2, 2.75)	0.999<br>-	2<br>(1.25, 2.75)	0.380<br>-	2<br>(1.25, 2)	0.327<br>-	2<br>(1.25, 2.75)	*0.008*<br>-
NV score								
qCSKs	0<br>(0, 0)	-<br>0.999	1<br>(1, 1.75)	-<br>0.291	2.5<br>(0.5, 3)	-<br>0.999	0.5<br>(0, 1.75)	-<br>*0.044*
aCSKs	0<br>(0, 0.75)	0.999/<br>0.999	1<br>(1, 1.75)	0.999/<br>0.157	2.5<br>(2, 3)	0.999/<br>0.999	1.5<br>(1, 2)	0.999/<br>0.313
SFs	0<br>(0, 0.75)	0.999/<br>0.999	1.5<br>(1, 2)	0.999/<br>0.999	2.5<br>(1.25, 3.75)	0.999/<br>0.999	3<br>(1.5, 3.75)	0.121/<br>0.999
Non-treated	0<br>(0, 0.75)	0.999<br>-	2<br>(2, 2.75)	0.291<br>-	3<br>(3, 3)	0.999<br>-	3.5<br>(3, 4)	*0.044*<br>-
Total score (haze + NV)								
qCSKs	2.5<br>(2, 3)	-<br>0.999	3<br>(2.25, 3)	-<br>0.146	3.25<br>(1.5, 3.5)	-<br>0.954	1<br>(0.5, 2.25)	-<br>*0.004*
aCSKs	2.5<br>(2, 3)	0.999/<br>0.999	3.5<br>(2.25, 4)	0.999/<br>0.393	3.25<br>(2.5, 4.75)	0.999/<br>0.999	2.25<br>(2, 2.875)	0.999/<br>*0.046*
SFs	2.5<br>(2, 3)	0.999/<br>0.999	4<br>(3.25, 4.75)	0.324/<br>0.999	4.5<br>(2.5, 6.5)	0.999/<br>0.999	4.5<br>(2.5, 5.75)	*0.031*/<br>0.999
Non-treated	3<br>(2.25, 3)	0.999<br>-	4.5<br>(3.25, 5)	0.146<br>-	4.5<br>(4, 5)	0.954<br>-	5<br>(4.25, 5.75)	*0.004*<br>-

Scores are expressed in median (Q1, Q3)

PID = post-injection day

*PID0 occurred 7 days after IrrPTK

NV = neovascularization

Source data are provided as a Source Data file

Supplementary Table 11. Corneal haze density (IntegratedDensity) following injections of different cell types in rat corneas with acute haze (n=6 in each group).

	PID0* (x10^6 arb. units)	p vs. qCSKs/ Non-treated	PID7 (x10^6 arb. units)	p vs. qCSKs/ Non-treated	PID14 (x10^6 arb. units)	p vs. qCSKs/ Non-treated	PID21 (x10^6 arb. units)	p vs. qCSKs/ Non-treated
qCSKs	12.40± 0.41	- 0.824	10.99± 1.05	- 0.275	9.02± 0.34	- *0.032*	8.19± 0.28	- *5.42x10^{-5}*
aCSKs	12.95± 0.38	0.766/ 0.999	12.13± 0.17	0.760/ 0.816	10.50± 0.26	0.591/ 0.330	9.49± 0.34	0.512/ *0.001*
SFs	12.45± 0.50	0.999/ 0.863	13.57± 0.66	0.149/ 0.983	11.51± 0.81	0.174/ 0.820	11.00± 0.44	*0.031*/ *0.047*
Non-treated	12.89± 0.27	0.824 -	13.15± 1.05	0.275 -	12.52± 1.36	*0.032* -	13.63± 1.16	*5.42x10^{-5}* -

PID = post-injection day

*PID0 occurred 7 days after IrrPTK

Source data are provided as a Source Data file

Supplementary Table 12. Percent corneal haze area following injections of different cell types in rat corneas with acute haze (n=6 in each group).

	PID0* (%)	p vs. qCSKs/ Non-treated	PID7 (%)	p vs. qCSKs/ Non-treated	PID14 (%)	p vs. qCSKs/ Non-treated	PID21 (%)	p vs. qCSKs/ Non-treated
qCSKs	38.03± 4.69	- 0.940	24.31± 4.13	- 0.603	11.64± 1.88	- 0.088	7.42± 1.09	- *0.045*
aCSKs	36.34± 5.13	0.995/ 0.987	24.07± 4.60	0.999/ 0.587	19.69± 3.75	0.740/ 0.458	21.63± 2.29	0.188/ 0.876
SFs	34.91± 6.06	0.972/ 0.999	36.49± 7.07	0.550/ 0.999	31.57± 4.48	0.086/ 0.999	36.49± 7.19	*0.002*/ 0.487
Non-treated	33.94± 4.25	0.940 -	35.69± 8.80	0.603 -	31.48± 9.31	0.088 -	26.70± 5.79	*0.045* -

PID = post-injection day

*PID0 occurred 7 days after IrrPTK

Source data are provided as a Source Data file

Supplementary Table 13. Percent change of central corneal thickness from preoperation following injections of different cell types in rat corneas with acute haze (n=6 in each group).

	PID0* (%)	p vs. qCSKs/ Non-treated	PID7 (%)	p vs. qCSKs/ Non-treated	PID14 (%)	p vs. CSKs/ Non-treated	PID21 (%)	p vs. qCSKs/ Non-treated
qCSKs	47.23±<br>8.48	-<br>0.999	10.45±<br>11.69	-<br>*0.036*	2.32±<br>7.75	-<br>0.085	-1.71±<br>5.72	-<br>0.238
aCSKs	48.70±<br>12.05	0.999/<br>0.999	6.17±<br>3.22	0.987/<br>*0.018*	1.57±<br>2.59	0.999/<br>0.073	3.57±<br>6.41	0.964/<br>0.468
SFs	48.79±<br>11.54	0.999/<br>0.999	6.20±<br>7.00	0.988/<br>*0.018*	3.54±<br>6.61	0.999/<br>0.108	8.40±<br>10.08	0.799/<br>0.727
Non-treated	48.15±<br>9.17	0.999<br>-	48.87±<br>11.89	*0.036*<br>-	27.13±<br>9.02	0.085<br>-	19.98±<br>8.42	0.238<br>-

PID = post-injection day

*PID0 occurred 7 days after IrrPTK

Source data are provided as a Source Data file

Supplementary Table 14. Synchrotron small-angle X-ray scattering outcomes in naïve rat corneas and corneas with acute haze following cell injections (n=9 in each group).

	IFD (nm)	p vs. naïve/ qCSKs	FD (nm)	p vs. naïve/ qCSKs	Matrix order (x10^6 arb. units)	p vs. naïve/ qCSKs
Naïve	72.0±0.3	-<br>*0.011*	32.5±0.3	-<br>*0.022*	1.75±0.10	-<br>0.804
qCSKs	76.8±1.4	*0.011*<br>-	33.6±0.3	*0.022*<br>-	1.86±0.13	0.804<br>-
SFs	73.7±0.7	0.647/<br>0.157	32.5±0.2	0.999/<br>*0.026*	1.37±0.06	*0.026*/<br>*0.003*
Non-treated	80.4±1.3	*1.09x10^{-5}*/<br>0.083	32.9±0.2	0.788/<br>0.168	1.32±0.05	*0.010*/<br>*0.001*

IFD = interfibrillar distance

FD = fibrillar diameter

Source data are provided as a Source Data file

Supplementary Table 15. Collagen fiber morphometric profiles of naïve rat corneas and corneas with acute haze following cell injections captured with multiphoton microscopy (n=3 in each group).

	Naïve	qCSKs	SFs	Non-treated
TAR (%)	29.56±0.05	29.73±1.90	21.57±2.35	22.69±0.67
p vs. naïve	-	0.999	*0.030*	0.061
p vs. qCSKs	0.999	-	*0.027*	0.055
CART (%)	91.58±0.04	88.02±0.90	79.78±2.35	79.72±0.81
p vs. naïve	-	0.384	*0.002*	*0.002*
p vs. qCSKs	0.384	-	*0.018*	*0.018*
CFD (%)	83.79±0.30	82.07±1.85	65.70±4.37	75.16±0.88
p vs. naïve	-	0.966	*0.006*	0.177
p vs. qCSKs	0.966	-	*0.010*	0.323
CARD	1.91±0.05	1.90±0.14	1.61±0.13	1.57±0.05
p vs. naïve	-	0.999	0.175	0.118
p vs. qCSKs	0.999	-	0.200	0.135
CFC (#/mm^2)	881±39	954±136	529±57	661±70
p vs. naïve	-	0.899	*0.044*	0.246
p vs. qCSKs	0.899	-	*0.017*	0.096
CFL (µm)	27.36±0.73	28.01±2.14	25.36±0.90	24.87±0.57
p vs. naïve	-	0.984	0.705	0.557
p vs. qCSKs	0.984	-	0.511	0.380

TAR = tissue area ratio

CART = collagen area ratio in tissue

CFD = collagen fiber density

CARD = collagen area reticulation density

CFC = collagen fiber count

CFL = collagen fiber length

Source data are provided as a Source Data file

Supplementary Table 16. Escape latency, distance traveled, and swim path efficiency in Morris water maze by naïve rats and laser-injured rats following cell injections (n=6 in each group).

	Naïve	qCSKs	SFs	Non-treated
Escape latency (s)	31.8±4.3	38.0±5.2	47.6±5.4	47.1±5.3
p vs. naïve	-	0.652	*0.030*	*0.036*
p vs. qCSKs	0.652	-	0.378	0.421
Distance traveled (m)	5.5±1.3	5.6±1.6	7.7±2.2	7.2±1.5
p vs. naïve	-	0.973	0.327	0.515
p vs. qCSKs	0.973	-	0.565	0.763
Path efficiency	0.31±0.09	0.28±0.10	0.22±0.09	0.22±0.08
p vs. naïve	-	0.975	0.722	0.700
p vs. qCSKs	0.975	-	0.913	0.899

Source data are provided as a Source Data file

Supplementary Table 17. Clinical haze and neovascularization scores following injections of different cell types in rat corneas with chronic haze (n=6 in each group).

	PID0*	p vs. qCSKs / Non-treated	PID7	p vs. qCSKs / Non-treated	PID14	p vs. qCSKs / Non-treated	PID21	p vs. qCSKs / Non-treated
Haze score								
qCSKs	2 (2, 2.75)	- 0.999	1 (1, 1.75)	- 0.216	0.75 (0.5, 1)	- *0.006*	1 (0.625, 1)	- *0.003*
SFs	2 (2, 2.75)	0.999/ 0.999	2 (2, 2)	0.216/ 0.999	2 (2, 2.75)	*0.019*/ 0.999	3 (2.25, 3)	*0.003*/ 0.999
Non-treated	2 (2, 2.75)	0.999 -	2 (2, 2)	0.216 -	2.5 (2, 3)	*0.006* -	2.5 (2, 3.75)	*0.003* -
NV score								
qCSKs	2.5 (2, 3)	- 0.999	2.5 (2, 3)	- 0.999	2 (2, 2)	- 0.299	2 (1.25, 2)	- *0.007*
SFs	2 (2, 2.75)	0.999/ 0.999	3 (3, 3)	0.999/ 0.999	3 (3, 3)	0.057/ 0.999	3.5 (3, 4)	*0.003*/ 0.999
Non-treated	2 (2, 2.75)	0.999 -	3 (3, 3)	0.999 -	3 (3, 3)	0.299 -	3 (3, 3.75)	*0.007* -
Total score (haze + NV)								
qCSKs	4.5 (4, 5.75)	- 0.999	3.5 (3, 4.75)	- 0.533	2.75 (2.5, 3)	- *0.021*	3 (1.875, 3)	- *0.001*
SFs	4.5 (4, 5)	0.999/ 0.999	5 (5, 5)	0.533/ 0.999	5 (5, 5.75)	*0.017*/ 0.999	6 (6, 6)	*0.001*/ 0.999
Non-treated	4.5 (4, 5)	0.999 -	5 (5, 5)	0.533 -	5.5 (5, 6)	*0.021* -	5.5 (5, 7.5)	*0.001* -

Scores are expressed in median (Q1, Q3)

PID = post-injection day

*PID0 occurred 28 days after IrrPTK

NV = neovascularization

Source data are provided as a Source Data file

Supplementary Table 18. Corneal haze density (IntegratedDensity) following injections of different cell types in rat corneas with chronic haze (n=6 in each group).

	PID0* ($x10^6$ arb. units)	p vs. qCSKs/ Non-treated	PID7 ($x10^6$ arb. units)	p vs. qCSKs/ Non-treated	PID14 ($x10^6$ arb. units)	p vs. qCSKs/ Non-treated	PID21 ($x10^6$ arb. units)	p vs. qCSKs/ Non-treated
qCSKs	16.40± 0.56	- 0.994	13.35± 1.02	- 0.894	11.64± 1.31	- 0.652	9.93± 0.52	- *0.001*
SFs	16.16± 0.66	0.954/ 0.919	14.27± 1.53	0.843/ 0.994	13.49± 0.97	0.417/ 0.914	13.67± 1.03	*0.006*/ 0.694
Non-treated	16.49± 0.51	0.994 -	14.09± 0.83	0.894 -	12.91± 0.64	0.652 -	14.52± 0.50	*0.001* -

PID = post-injection day

*PID0 occurred 28 days after irrPTK

Source data are provided as a Source Data file

Supplementary Table 19. Percent corneal haze area following injections of different cell types in rat corneas with chronic haze (n=6 in each group).

	PID0* (%)	p vs. qCSKs/ Non-treated	PID7 (%)	p vs. qCSKs/ Non-treated	PID14 (%)	p vs. qCSKs/ Non-treated	PID21 (%)	p vs. qCSKs/ Non-treated
qCSKs	36.23± 5.00	- 0.820	28.35± 4.27	- 0.434	22.67± 4.54	- 0.069	28.37± 7.35	- 0.068
SFs	36.74± 2.58	0.996/ 0.772	57.04± 4.50	*0.008*/ 0.089	55.36± 2.81	*0.001*/ 0.085	67.55± 2.50	*0.001*/ 0.099
Non-treated	32.78± 4.17	0.820 -	38.59± 7.69	0.434 -	39.42± 6.54	0.069 -	48.83± 6.77	0.068 -

PID = post-injection day

*PID0 occurred 28 days after IrrPTK

Source data are provided as a Source Data file

Supplementary Table 20. Percent change of central corneal thickness from preoperation following injections of different cell types in rat corneas with chronic haze (n=6 in each group).

	PID0* (%)	p vs. qCSKs/ Non-treated	PID7 (%)	p vs. qCSKs/ Non-treated	PID14 (%)	p vs. CSKs/ Non-treated	PID21 (%)	p vs. qCSKs/ Non-treated
qCSKs	8.02± 9.84	- 0.940	2.46±12 .07	- 0.450	-4.76± 8.71	- 0.850	-2.85± 8.49	- 0.987
SFs	28.07± 17.21	0.259/ 0.413	32.97± 7.75	0.104/ 0.613	18.47± 9.93	0.161/ 0.366	13.92± 7.97	0.253/ 0.317
Non-treated	12.11± 8.66	0.940 -	19.65±9 .13	0.450 -	1.75± 6.31	0.850 -	-1.29 ±4.21	0.987 -

PID = post-injection day

*PID0 occurred 28 days after IrrPTK

Source data are provided as a Source Data file

Supplementary Table 21. Cornea donors' information.

S/N	Age	Sex	Days to culture	Cause of death
1	29	M	16	Intracerebral hemorrhage
2	72	M	14	End-stage liver disease
3	15	F	9	Trauma
4	47	M	14	Peritonitis
5	29	M	13	Cerebral herniation and glioblastoma
6	21	F	10	Blunt traumatic injuries
7	60	F	15	Septic shock
8	42	M	14	Ruptured middle cerebral artery aneurysm
9	65	F	13	Probable myocardial infarction
10	55	F	14	Coronary artery disease
11	35	F	16	Pulmonary and cerebral edema
12	18	M	12	Multi-vehicle accident
13	60	F	11	Myocardial Infarction
14	45	F	11	Myocardial Infarction

Supplementary Table 22. Primer sequence information.

Gene (human)	GenBank accession no.	Sequence
ALDH1A1	NM_000689	F: CGTTGGTTATGCTCATTTGGAAGA<br>R: CTTTTGATCACGTCATCTAAAGAT
ALDH3A1	NM_001135168	F: CATTGGCACCTGGAACTACC<br>R: GGCTTGAGGACCACTGAGTT
LUM	NM_002345	F: CCTGGTTGAGCTGGATCTGT<br>R: TGGTTTCTGAGATGCGATTG
KERA	NM_007035	F: ATCTGCAGCACCTTCACCTT<br>R: CATTGGAATTGGTGGTTTGA
CHST6	NM_021615	F: TACCGGCCTGTGTACTCTGA<br>R: ACTAATTTCGGGGGTGCGAG
B3GNT7	NM_145236	F: AGTCTCACCCCTGGTCAGTT<br>R: AGCAGTTAGTGGTGGTCACG
COL8A2	NM_005202	F: ACATCCAGCCCATGCAGAAA<br>R: GCATTTCCAGGTACTGGCCT
