# Supplementary material for: Impact of keratocyte differentiation on corneal opacity resolution and visual function recovery in male rats
Source: Nat Commun. 2024 Jun 11;15:4959. doi: 10.1038/s41467-024-49008-3 (PMC11166667; doi:10.1038/s41467-024-49008-3)
Supplement: Supplementary file 3 — Description of Additional Supplementary Files [file 41467_2024_49008_MOESM3_ESM.pdf]

Description of Additional Supplementary Files

Supplementary Movie 1. Representative video showing good quality cell delivery into the stroma.

Supplementary Movie 2. Representative video showing one of the characteristics of poor cell delivery: Multiple needle entry point (tunnel collapsed due to soft epithelium in some corneas, requiring the creation of another injection point).

Supplementary Movie 3. Representative video showing one of the characteristics of poor cell delivery: Decentered delivery of cells.

Supplementary Movie 4. Representative video showing one of the characteristics of poor cell delivery: Backflow of injected cells, reducing the number of cells at the therapy site.
