# Supplementary material for: Impact of keratocyte differentiation on corneal opacity resolution and visual function recovery in male rats
Source: Nat Commun. 2024 Jun 11;15:4959. doi: 10.1038/s41467-024-49008-3 (PMC11166667; doi:10.1038/s41467-024-49008-3)
Supplement: Supplementary file 8 — Reporting Summary [file 41467_2024_49008_MOESM8_ESM.pdf]

nature portfolio

Corresponding author(s): Jodhbir Mehta

Last updated by author(s): Apr 10, 2024

Reporting Summary

Nature Portfolio wishes to improve the reproducibility of the work that we publish. This form provides structure for consistency and transparency in reporting. For further information on Nature Portfolio policies, see our Editorial Policies and the Editorial Policy Checklist.

Statistics

For all statistical analyses, confirm that the following items are present in the figure legend, table legend, main text, or Methods section.

n/a	Confirmed	
☐	☒	The exact sample size (*n*) for each experimental group/condition, given as a discrete number and unit of measurement
☐	☒	A statement on whether measurements were taken from distinct samples or whether the same sample was measured repeatedly
☐	☒	The statistical test(s) used AND whether they are one- or two-sided<br>*Only common tests should be described solely by name; describe more complex techniques in the Methods section.*
☒	☐	A description of all covariates tested
☐	☒	A description of any assumptions or corrections, such as tests of normality and adjustment for multiple comparisons
☐	☒	A full description of the statistical parameters including central tendency (e.g. means) or other basic estimates (e.g. regression coefficient) AND variation (e.g. standard deviation) or associated estimates of uncertainty (e.g. confidence intervals)
☐	☒	For null hypothesis testing, the test statistic (e.g. F, t, r) with confidence intervals, effect sizes, degrees of freedom and P value noted<br>*Give P values as exact values whenever suitable.*
☒	☐	For Bayesian analysis, information on the choice of priors and Markov chain Monte Carlo settings
☒	☐	For hierarchical and complex designs, identification of the appropriate level for tests and full reporting of outcomes
☒	☐	Estimates of effect sizes (e.g. Cohen's *d*, Pearson's *r*), indicating how they were calculated

Our web collection on statistics for biologists contains articles on many of the points above.

Software and code

Policy information about availability of computer code

Data collection	No software was used.
Data analysis	Quantification of haze with slit-lamp and IVCM images were carried out with ImageJ version 1.53t. The central corneal thickness was measured with software built in the AS-OCT. The SAXS data was analyzed with SAXS4COLL software, which was detailed in J. Appl. Crystallogr. 50, 1235-1240 (2017). The multiphoton microscopy data was analyzed with software that was included in the microscope.

For manuscripts utilizing custom algorithms or software that are central to the research but not yet described in published literature, software must be made available to editors and reviewers. We strongly encourage code deposition in a community repository (e.g. GitHub). See the Nature Portfolio guidelines for submitting code & software for further information.

Data

Policy information about availability of data

All manuscripts must include a data availability statement. This statement should provide the following information, where applicable:

- Accession codes, unique identifiers, or web links for publicly available datasets
- A description of any restrictions on data availability
- For clinical datasets or third party data, please ensure that the statement adheres to our policy

All raw values used to generate the graphs are included in the Source Data file. The entire raw dataset of animal experiments (corneal imaging) is more than 4 TB in size and can be shared on request with appropriate data-transfer methods.

Research involving human participants, their data, or biological material

Policy information about studies with human participants or human data. See also policy information about sex, gender (identity/presentation), and sexual orientation and race, ethnicity and racism.

Reporting on sex and gender	Not applicable. This study did not involve human research participants.
Reporting on race, ethnicity, or other socially relevant groupings	Not applicable.
Population characteristics	Not applicable.
Recruitment	Not applicable.
Ethics oversight	Not applicable.

Note that full information on the approval of the study protocol must also be provided in the manuscript.

Field-specific reporting

Please select the one below that is the best fit for your research. If you are not sure, read the appropriate sections before making your selection.

☒ Life sciences ☐ Behavioural & social sciences ☐ Ecological, evolutionary & environmental sciences

For a reference copy of the document with all sections, see nature.com/documents/nr-reporting-summary-flat.pdf

Life sciences study design

All studies must disclose on these points even when the disclosure is negative.

Sample size	No sample size calculation was performed. All animal experiments that require corneal haze and vascularization grading were represented by at least 6 rats. The rest of the experiments, e.g., qPCR, SAXS, immunofluorescence staining, TEM, and multiphoton microscopy, were conducted on at least 3 independent samples. These numbers were derived on our previous experience with studying cell therapy in the rats. The study was published in Invest. Ophthalmol. Vis. Sci. 59, 3340-3354 (2018).
Data exclusions	No data was excluded.
Replication	All experiments were carried out on at least 3 independent samples. The n number was included in the Methods, figure legends, and table legends.
Randomization	Rats were randomized for cell therapies.
Blinding	The surgeon, who performed the surgery in the rats, was masked from the cell type that he injected. To avoid further bias, the grader was also masked when grading the degree of corneal haze and vascularization.

Reporting for specific materials, systems and methods

We require information from authors about some types of materials, experimental systems and methods used in many studies. Here, indicate whether each material, system or method listed is relevant to your study. If you are not sure if a list item applies to your research, read the appropriate section before selecting a response.

Materials & experimental systems

n/a	Involved in the study	
☐	☒	Antibodies
☐	☒	Eukaryotic cell lines
☒	☐	Palaeontology and archaeology
☐	☒	Animals and other organisms
☒	☐	Clinical data
☒	☐	Dual use research of concern
☒	☐	Plants

Methods

n/a	Involved in the study	
☒	☐	ChIP-seq
☒	☐	Flow cytometry
☒	☐	MRI-based neuroimaging

Antibodies

Antibodies used

For staining human cells:
Keratocan, Atlas Antibodies, cat. no. HPA039321
Lumican, Bioss, cat. no. bs-5890R
ALDH3A1, Proteintech, cat. no. 15578-1-AP

For staining rat tissues:
Thy-1 (clone OX-7), Novus Biologicals, cat. no. NB100-65543
Fibronectin (clone DH1), Sigma-Aldrich, cat. no. MAB1940
Keratan sulfate (clone 5D4), MyBioSource, cat. no. MBS442009
Keratocan, MyBioSource, cat. no. MBS9462578
Lumican (clone JE11-45), HUABIO, cat. no. ET7109-24
α-SMA (clone 1A4), Agilent Technologies, cat no. M0851
Collagen 3A1 (clone 1E7-D7/Col3), Novus Biologicals, cat. no. NBP1-05119

Secondary antibodies:
Goat anti-rabbit Red-X, Jackson ImmunoResearch Laboratories, cat. no. 111-295-003
Goat anti-mouse Red-X, Jackson ImmunoResearch Laboratories, cat. no. 115-295-003
Goat anti-mouse Alexa Fluor 488, Jackson ImmunoResearch Laboratories, cat. no. 115-545-003

Validation

The antibodies were selected based on the matching species reactivity (human or rat) in the datasheets. The majority of the antibodies have also been used in our previous publications in Cell Transplant. 24, 1845-1861 (2015) and Invest. Ophthalmol. Vis. Sci. 59, 3340-3354 (2018) for staining human or rat samples.

Eukaryotic cell lines

Policy information about cell lines and Sex and Gender in Research

Cell line source(s)

Primary corneal stromal cells (qCSKs, aCSKs, and SFs) were derived from research-grade cadaveric corneas deemed unsuitable for transplantation were procured from Lions World Vision Institute (Tampa, FL) and Saving Sight (Kansas City, MO). The sex of the donors (6 males and 8 females) was included in Supplementary Table 21.

Authentication

Cells were authenticated by morphological appearance and keratocan gene expression.

Mycoplasma contamination

Not tested.

Commonly misidentified lines
(See ICLAC register)

Not applicable.

Animals and other research organisms

Policy information about studies involving animals; ARRIVE guidelines recommended for reporting animal research, and Sex and Gender in Research

Laboratory animals

Rats (Rattus norvegicus), Sprague-Dawley strain, 6-8 weeks old.

Wild animals

No wild animals were used.

Reporting on sex

We only used male rats. A total of 120 rats were used in the study. To the best of our knowledge, there is no evidence that rat sex has a differential effect on the corneal stromal wound healing response.

Field-collected samples

No field-collected samples were used.

Ethics oversight

The animal experiments were performed in accordance with The ARVO Statement for the Use of Animals in Ophthalmic and Vision Research and approved by the Institutional Animal Care and Use Committee of SingHealth.

Note that full information on the approval of the study protocol must also be provided in the manuscript.
